# Salt-Dependent Chemotaxis of Macrophages

**DOI:** 10.1371/journal.pone.0073439

**Published:** 2013-09-16

**Authors:** Silke Müller, Thomas Quast, Agnes Schröder, Stephanie Hucke, Luisa Klotz, Jonathan Jantsch, Rupert Gerzer, Ruth Hemmersbach, Waldemar Kolanus

**Affiliations:** 1 Institute of Aerospace Medicine, German Aerospace Center, Cologne, Germany; 2 Laboratory of Molecular Immunology, Life and Medical Sciences (LIMES) Institute, University of Bonn, Bonn, Germany; 3 Interdisciplinary Center for Clinical Research, Friedrich-Alexander-University Erlangen-Nuremberg, Erlangen, Germany; 4 Clinic for Neurology – Inflammatory Disorders of the Central Nervous System and Neurooncology, University of Münster, Münster, Germany; 5 Microbiology Institute – Clinical Microbiology, Immunology and Hygiene, University Hospital of Erlangen and Friedrich-Alexander-University Erlangen-Nuremberg, Erlangen, Germany; Chinese University of Hong Kong, Hong Kong

## Abstract

Besides their role in immune system host defense, there is growing evidence that macrophages may also be important regulators of salt homeostasis and blood pressure by a TonEBP-VEGF-C dependent buffering mechanism. As macrophages are known to accumulate in the skin of rats fed under high salt diet conditions and are pivotal for removal of high salt storage, the question arose how macrophages sense sites of high sodium storage. Interestingly, we observed that macrophage-like RAW264.7 cells, murine bone marrow-derived macrophages and peritoneal macrophages recognize NaCl hypertonicity as a chemotactic stimulus and migrate in the direction of excess salt concentration by using an *in vitro* transwell migration assay. While RAW264.7 cells migrated toward NaCl in a dose-dependent fashion, no migratory response toward isotonic or hypotonic media controls, or other osmo-active agents, e.g. urea or mannitol, could be detected. Interestingly, we could not establish a specific role of the osmoprotective transcription factor TonEBP in regulating salt-dependent chemotaxis, since the specific migration of bone marrow-derived macrophages following RNAi of TonEBP toward NaCl was not altered. Although the underlying mechanism remains unidentified, these data point to a thus far unappreciated role for NaCl-dependent chemotaxis of macrophages in the clearance of excess salt, and suggest the existence of novel NaCl sensor/effector circuits, which are independent of the TonEBP system.

## Introduction

Macrophages are motile hematopoietic cells that play important roles in immune surveillance by secreting cytokines or by phagocytosing pathogens as well as apoptotic cells [1]. Recent studies support the hypothesis that macrophages are not only essential for efficient immune responses, but are also regulators of an extrarenal salt balance system, which controls blood pressure [2].

Results from human spaceflight raised questions about salt homeostasis and indicated a novel mechanism of salt storage without water retention [3]. It has been demonstrated that Na^+^ can be stored in the skin in abundance over water, creating a local electrolyte environment that does not readily equilibrate with plasma and hence escapes control of renal blood purification [4]. Macrophages infiltrate the skin of rodents following high salt-diet, suggesting that they may control the electrolyte homeostasis of this compartment [2]. It was shown that this buffering mechanism depends on a transcription factor termed tonicity enhancer binding protein (TonEBP), which directs vascular endothelial growth factor C (VEGF-C) driven hyperplasia of the lymph capillary network. Blockade of this regulatory axis resulted in skin electrolyte accumulation and blood pressure increase [2]. Furthermore, the discovered mechanism may be active in humans as well: in recent studies on salt-sensitive hypertension, elevated VEGF-C levels were found in the serum of patients with high blood pressure [2], [5].

We thus hypothesized that macrophages migrate chemotactically toward high sodium concentrations in areas of salt storage. Although recruitment of macrophages and monocytes into skin tissue by chemotaxis plays a crucial role in immune functions, we investigated whether NaCl-mediated hypertonic stress acts as chemotactic stimulus per se and demonstrate here for the first time robust migratory responses of RAW264.7 macrophages, murine bone marrow-derived macrophages and murine peritoneal macrophages toward different NaCl gradients in a transwell migration assay *in vitro* (modified Boyden chamber). We also assessed the role of the osmoprotective transcription factor TonEBP in salt-dependent chemotaxis by analyzing migration behavior of RAW264.7 cells with constitutive TonEBP overexpression and RNA interference (RNAi) of TonEBP. Although TonEBP is a key regulator in the removal of excess salt *in vivo*, it is not regulating salt-dependent chemotaxis of macrophages. This suggests the existence of alternative sensors for excess NaCl in macrophages.

## Materials and Methods

### Ethic Statement

All animal experiments were conducted in a licensed animal facility in accordance with the German law on the protection of experimental animals and were approved by local authorities of the state of Nordrhein-Westfalen (Landesamt für Natur, Umwelt und Verbraucherschutz NRW). Mice were sacrified by cervical dislocation.

### Cell culture

The murine macrophage cell line RAW264.7 (#TIB-71) was purchased from ATTC. RAW264.7 macrophages with a stable TonEBP overexpression were generated as described earlier by Machnik et al [2]. Cells were cultured in Dulbeccós modified Eaglés Medium (DMEM) (PAA) supplemented with 10% Fetal Bovine Serum (Biochrom, Berlin, Germany) and 1% Pen-Strep (Biochrom) in a 5% CO_2_ atmosphere with 95% humidity at 37°C.

Bone marrow-derived dendritic cells (BMDCs) were generated as described previously by Quast et al. [6]. In brief, bone marrow was harvested from the femurs and tibiae of seven week old C57BL/6 wildtype and filtered with 40 μm pore nylon cell strainers (BD Biosciences). Then, hematopoietic stem cells were plated into 10 cm non-treated petri dishes at a concentration of 5*10^6^ cells in 10 mL VLE-RPMI 1640 (Biochrom) supplemented with 10% vol/vol heat-inactivated FCS (Sigma-Aldrich), 100 u/mL Penicillin (PAA), 0.1 mg/mL Streptomycin (PAA) and 10 ng/mL recombinant murine GM-CSF (Peprotech). The culture medium was half-renewed every three days. At day 7–10 of culture, BMDCs were stimulated to mature by adding 200 ng/mL LPS (derived from *E. coli*, Sigma-Aldrich) for 48 h before they were used for functional assays.

For differentiation into bone marrow-derived macrophages (BMDMs), cells were plated into 10 cm non-treated petri dishes at a concentration of 5*10^6^ cells in IMDM medium (PAA) supplemented with 10% FCS (Sigma-Aldrich), 2 mM L-glutamine (PAA), 100 u/mL Penicillin (PAA), 0.1 mg/mL Streptomycin (PAA) and 10 ng/mL recombinant murine M-CSF (Peprotech).

Peritoneal macrophages were isolated via peritoneal lavage of C57BL/6 mice with 2 mM EDTA/PBS. After centrifugation at 1000 rpm for 8 min, remaining erythrocytes were lysed in erythrocyte lysis buffer (150 mM NH_4_Cl, 10 mM KHCO_3_ in ddH_2_O) for 5 min at 37°C. The reaction was stopped with 35 ml ice-cold PBS and washed twice with PBS following centrifugation at 1000 rpm for 5 min. Then, cells were plated into 10 cm non-treated petri dishes in IMDM supplemented with 1% Pen-Strep and 10% FCS. After adhesion of the macrophages, the cell culture medium was replaced in order to remove remaining B-cells in the supernatant.

### *In vitro* migration assay

Chemotaxis of the macrophage cell line RAW264.7, bone marrow-derived macrophages and peritoneal macrophages was analyzed with a modified Boyden chamber (transwell) assay using cell culture membrane inserts with 8 µm pore size (BD Falcon #353097, Becton Dickinson). 2*10^5^cells were placed in serum-reduced (0.5% FCS) cell culture media (see cell culture) in the upper well while the culture medium of the lower compartment was supplemented with 25 nM CXCL12, 15 nM CCL2 (both from Peprotech), or NaCl (Merck) with concentrations between 10–100 mM (reaching a final concentration of 155 to 255 mM NaCl in the media), respectively. After 20 hours non-migratory cells on top of the membrane were removed with cotton swabs before the transmigrated cells on the bottom of the membrane were stained with 5 µM Vybrant CFDA-SE in PBS (Invitrogen) according to the manufacturer’s protocol. For each sample, 5–10 random fields were observed with an inverted Nikon Eclipse TE 2000-E fluorescence microscope (Nikon), equipped with a PlanFluor DL 10x/0.30 N.A. objective (Nikon). The number of migrated cells was counted using Cell Profiler software as described in [7].

For migration analysis of LPS-activated BMDCs, 20 nM CCL19 (Peprotech) and cell culture inserts with 5 µm pore size were used (Corning). Transmigrated BMDCs in the lower compartment were counted after incubation for 4 h at 37°C/5% CO_2_.

### Cell viability test

1*10^4^ cells per well were placed in a 96 well plate in serum-reduced media (0.5% FCS) to adhere. Subsequently, Na^+^ concentration of the media was increased by adding 10 to 100 mM NaCl. After 20 hours, the metabolic activity of the cells referring to cell viability was tested using a Cell Titer Blue Assay (Promega) according to manufacturer’s protocol. Fluorescence output of the assay was measured with a GloMax-Multi microplate reader (Promega).

### Osmometer measurements

Osmolality of media solutions following different osmotic stimuli was determined with the freezing point micro-osmometer OM815 (Vogel Löser). A 300 mosm/kg H_2_O standard solution was used to calibrate the osmometer and accuracy of measurements was ensured by using a sample of 0.9% NaCl with known osmolality of 186 mosm/kg H_2_O.

### Flow cytometry

Activation of BMDM macrophages was analyzed using flow cytometry (FACS CantoII device, BD Biosciences) following staining with an PE-conjugated anti-CD86 antibody (eBioscience).

### Flame photometry

Media samples of both the upper and the lower chambers of the transwell system were collected at different time points after NaCl supplementation (1, 10, 30 minutes, and 1, 2, 4, 8, 12, 16 and 20 hours). Na^+^ content in the supernatants was determined via flame photometry in an EFOX 5053 device (Eppendorf) by comparison with an urine standard (EFK diagnostics). Liquichek Urine Chemistry Control (Biorad) containing known Na^+^ concentrations was used to ensure the accuracy of the photometer.

### Phalloidin staining

5*10^5^ RAW264.7 cells were placed in DMEM with reduced FCS (0.5%) on fibronectin-coated (Bio-Harbor) glass coverslips. Cells were stimulated with 40 mM NaCl (Merck), 10 nM C5a (Peprotech), 15 nM CCL2 (Peprotech) or 25 nM CXCL12 (Peprotech) for 5 min. Subsequently, coverslips were washed twice with PBS followed by fixation with 4% PFA (Roth) in PBS for 20 minutes. Permeabilization of the cells was performed with 0.2% TritonX100 (Roth) in PBS for 5 minutes. Cells were stained with TRITC-Phalliodin (Sigma-Aldrich) and DAPI (Sigma-Aldrich) for 45 min. After washing three times with PBS and one time with bidest, coverslips were mounted in FluoroShield (ImmunoBioscience Corp.) containing 50 mg/ml DABCO anti-fade reagent (Sigma-Aldrich). Cell morphology and actin cytoskeleton organization was analyzed by the use of an inverted confocal laser scanning microscope (Fluoview 1000, Olympus) equipped with a Plan Apochromat 60x, NA 1.4 oil immersion objective (Olympus).

### Analysis of lamellipodia dynamics

Motile RAW264.7 cells were placed in DMEM (PAA) with reduced FCS (0.5%) on a glass surface of a Lab-Tek Chamber Slide (Nunc) to adhere for 20 min in a 5% CO_2_ atmosphere with 95% humidity at 37°C. Subsequently, cells were stimulated with 40 mM NaCl (Merck), 10 nM C5a (Peprotech), 15 nM CCL2 (Peprotech) or 25 nM CXCL12 (Peprotech), respectively. To analyze lamellipodia dynamics of macrophages, RAW264.7 cells were monitored over a period of 5 min by capturing phase contrast images every 2 s using an inverted Nikon Eclipse TE 2000-E microscope equipped with a climate chamber (37°C, 5% CO_2_, humidity) and a PlanApo DM 100x/1.40 N.A. oil immersion phase contrast objective (Nikon). Subsequently, an area of interest was marked on each image of the time-series by lines (white line) that cross the motile lamellipodium of the polarized cell. The resulting 1-pixel-wide areas were cut out and lined up in time space plots. In these time space plots (so-called kymographs) lamellipodia protrusions appear as linear ascending contours (see yellow lines). The slope of these lines corresponds to the velocity of lamellipodia movement, v = dx (µm)/dt (min). Velocities of membrane protrusions were analyzed by kymograph and line-scan analysis following the walking average plug-in, using ImageJ1.38 (National Institutes of Health; http://imagej.nih.gov/ij/).

### Western blot

For analysis of TonEBP protein expression, 2*10^6^ RAW264.7 cells were plated in cell culture dishes in serum reduced DMEM (0.5% FCS) and exposed to additional 40 mM NaCl for 20 hours. Afterwards, cells were washed with PBS and lysed in 8 M urea (Roth) with protease inhibitor (Roche Applied Science), followed by an incubation for 10 min on ice and centrifugation for 10 min at 13.000 rpm. The supernatant was used for immunoblot analysis and the protein concentration was measured by Bradford assay using Roti-Quant (Roth).

For immunoblotting, equal amounts of total protein were separated on 8% SDS-polyacrylamide gels under reducing conditions and electroblotted onto a polyvinylidene diflouride (PVDF) membrane (Biorad). The blots were blocked with 5% nonfat milk in phosphate buffered saline (PBS) and 0.1% Tween 20, pH 7.5 for 1 h at room temperature and then incubated overnight at 4°C with an anti-TonEBP antibody (ABR) and anti-β-actin (Sigma-Aldrich), respectively, both diluted 1:1.000 in 5% nonfat milk in PBST. After washing in PBST for three times, the blots were incubated with horseradish peroxidase-conjugated anti-rabbit IgG (Pierce) diluted 1:1000 in blocking solution for 90 minutes at room temperature. After washing in PBST for three times, chemiluminescent signals were detected using ECL Western Blotting Substrate (Pierce) on autoradiographic film (Fujifilm).

### RNA interference of TonEBP

3*10^5^ RAW264.7 cells were placed into 6-well plates and transfected using Lipofectamine 2000 Transfection Reagent (Invitrogen) on the next day according to the manufactureŕs protocol. Prior to transfection, we replaced the medium with serum-free medium without antibiotics and diluted 100 pmol of siRNA duplexes against the target sequence of TonEBP (Dharmacon; prevalidated siRNA database catalog L 058868) or 100 pmol of AllStars Negative siRNA (Qiagen catalog 1027281) in 250 µl Opti-MEM (Invitrogen), respectively. After incubation for 4 hours at 37°C/5% CO_2_, cells were washed and the medium was replaced with normal DMEM. Three days after transfection cells were used for functional migration assays. Efficiency of the transfection and RNAi was evaluated by Western Blot analysis after stimulation with additional 40 mM NaCl for 20 hours.

### Detection of CCL2 production

2*10^5^ RAW264.7 cells were placed in a 96 well plate in serum reduced DMEM (0.5% FCS) and stimulated with additional 40 mM NaCl. After 1, 2, 4, 8, 12, 16 and 20 hours, supernatants of NaCl stimulated cells and supernatants of cells cultivated in control medium (DMEM +0.5% FCS) were collected. Murine CCL2 in supernatants was quantified at the indicated timepoints by ELISA, according to the manufacturer`s instruction (R&D Duoset).

### Statistical analysis

All experiments were repeated at least three times using duplicates. Data are expressed as mean ± SD. Two-tailed Student’s t test with values of p<0.05 considered significant was performed with IBM SPSS Statistics 19.0 (IBM) software.

## Results

### NaCl induces chemotaxis of RAW264.7 cells

A recent study demonstrates that macrophages, which infiltrate the interstitium of the skin, are able to regulate salt-dependent extracellular volume and blood pressure homeostasis by a TonEBP-VEGF-C-dependent buffering mechanism [2]. However, it remained unclear whether hypertonic stress and NaCl act as a chemotactic stimulus per se. To investigate this issue we analyzed the ability of RAW264.7 macrophages to actively migrate toward excess NaCl by the use of modified Boyden chamber assays (so-called transwell migration assays).

**Figure 1:**
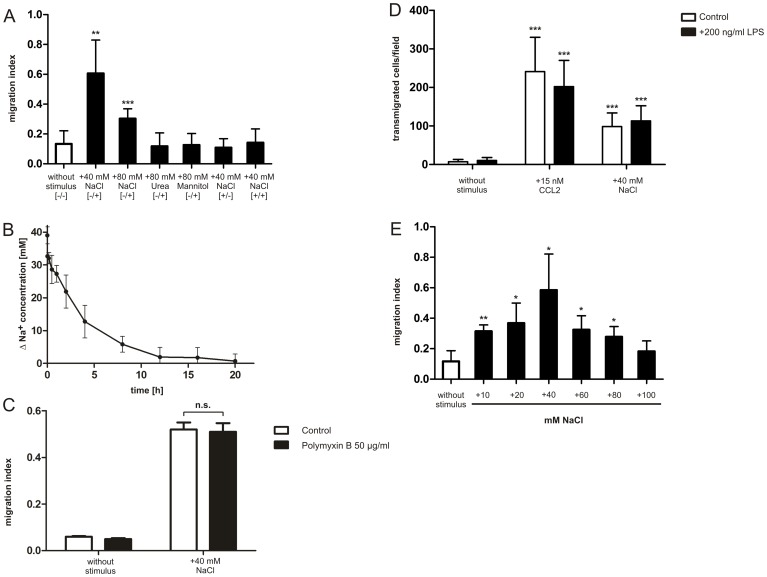
RAW264.7 cells recognize NaCl-induced hypertonicity as a chemoattractant stimulus. Transwell migration assay of the murine macrophage cell line RAW264.7 toward the hypertonic stimuli NaCl, urea or mannitol, respectively (A). The difference in Na^+^ concentration measured in lower and upper wells by flame photometry is shown as Δ Na^+^ (B). Transwell migration assay of RAW264.7 cells in the presence or absence of Polymyxin B, respectively (C). Transwell migration assay of BMDMs activated with 200 ng/ml LPS (D). Dose-dependency of RAW264.7 cells toward different NaCl concentrations by the use of transwell migration assays (E). The migration index was determined by the number of transmigrated cells in relation to CXCL12-stimulated cells after 20 hours. [−/−] indicates that no hypertonic stimulus was added to the transwells. [−/+] indicates that the hypertonic stimulus was added to the lower well of the transwell. [+/−] indicates that the hypertonic stimulus was added to the upper well of the transwell. [+/+] indicates that the hypertonic stimulus was added to both wells of the transwell (A). “+”indicates that the stimulus was added to the lower well of the transwell (C, D and E). Error bars indicate ± SD. *p<0.05, **p<0.01, ***p<0.001 as compared to control of cells without stimulus. n.s. not significant. n>3 in duplicates.

The NaCl concentration in the lower well was increased by 40 mM (to a final NaCl concentration of 195 mM). This sodium concentration has been established previously by Machnik et al. to simulate the difference between the combined serum Na^+^ and K^+^ concentrations and the ratio of the Na^+^ and K^+^ content relative to water in skin observed in rats fed on a high salt diet [2]. Quantification of chemotaxis after 20 hours revealed a highly significant increase of transmigrated RAW264.7 cells toward excess NaCl as compared to unstimulated control cells (Fig. 1a). Migration capacity of NaCl-stimulated cells was about 60% as compared to cells stimulated with the chemokine CXCL12 (Fig. 1a). We found a lower but still significantly enhanced cell migration response toward 80 mM NaCl. However, the control osmolytes urea or mannitol were unable to elicit a significant chemotactic response of RAW264.7 cells (Fig. 1a), therefore illustrating that the observed cell migration was specific to increased hypertonicity by NaCl but not osmolality in general, or hypertonicity by mannitol. Indeed, cryoscopic (freezing point) measurements confirmed that the osmolality of the applied hypertonic solutions was equivalent and resulted in an increase from 320 mosm/kg H_2_O in culture medium to approximately 400 mosm/kg H_2_O following application of NaCl, urea or manntitol, respectively (Table S1).

We then asked whether the observed induction of migration of RAW264.7 cells toward NaCl was due to a gradient-independent activation by a uniformly applied high salt concentration (so-called chemokinesis), and performed transwell migration assays with 40 mM excess NaCl in the upper well (+/−) or in both wells (+/+), respectively (Fig. 1a). In contrast to the setting in which NaCl was added to the lower well, we could not detect enhanced directional migration of RAW264.7 cells under these conditions. Taken together, these data provide strong evidence that a NaCl concentration gradient is a prerequisite for salt-induced chemotaxis of RAW264.7 cells.

Since our data suggested that concentration gradients of sodium and chloride ions are important for NaCl induced chemotaxis of RAW264.7 cells (see Fig. 1a), we assessed the stability of the NaCl gradient during the course of the transwell assay. For this reason we measured the Na^+^ concentration in the upper and lower wells of the transwell chambers with the help of a flame photometer. By comparing the Na^+^ content in the media with an urine standard (143.5 mmol/L), we found that the Na^+^ concentration in the lower well started to decrease rapidly from the 40 mM NaCl at t = 0, but a differential of at least 10 mM Na^+^ was still detectable for approximately 8 hours (Fig. 1c). We thus conclude that NaCl gradients can form and persist in this system and are likely responsible for the observed biological responses.

Previous reports have shown that motility of macrophages is enhanced when the cells are activated by endotoxins [8]. To exclude that migration of the cells toward high NaCl concentrations is due to a contamination by Lipopolysaccharide (LPS) followed by activation of macrophages, we incubated all media solutions with 50 µg/ml polymyxin B (PmxB). This concentration was able to attenuate nitric oxide production in RAW264.7 macrophages following stimulation with 1 µg/ml LPS without altering cell viability (data not shown). Analysis of the migration behavior revealed no significant difference between cells migrating in the presence or absence of PmxB (Fig. 1d). We conclude from these findings that NaCl-stimulated chemotaxis of RAW264.7 cells could not be attributed to a LPS-dependent inflammatory response. This is supported by the finding that LPS-activated, CD86- positive BMDMs (Fig. S1) show no random motility in the absence of a chemotactic stimulus (Fig. 1d). Furthermore, we found that the migration capacity of macrophages toward CCL2 or NaCl is unaltered following LPS-maturation as compared to unstimulated BMDMs.

Because we found a higher migration rate of RAW264.7 cells toward 40 mM excess NaCl as compared to 80 mM excess NaCl (see Fig. 1a) we sought to determine the maximum motility response toward NaCl by employing a dose-dependent transwell migration assay. Upon increasing excess NaCl concentrations (0 to 100 mM) in the lower well, we observed that the migratory responses of the cells toward NaCl were dose-dependent and showed a maximum at approximately 40 mM excess NaCl (Fig. 1e). The same NaCl concentrations were tested in a cell titer blue metabolic viability assay to investigate whether the decreased migratory response at concentrations higher than 40 mM excess NaCl is due to reduced cell viability. After 20 h exposure, cells showed more than 90% viability in added NaCl concentrations up to 40 mM. Nevertheless, cell viability slightly but significantly decreased from additional 60 mM NaCl onward (Fig. S2). We conclude from these data that decreased migration is likely caused by reduced viability of the cells at concentrations higher than 40 mM excess NaCl. Therefore, in all following experiments a stimulus of 40 mM excess NaCl was used, since this concentration elicited the maximum migration response, had no effect on cell viability and corresponded to *in vivo* data described previously [2].

### Bone marrow derived macrophages, but not dendritic cells, migrate toward excess NaCl

**Figure 2:**
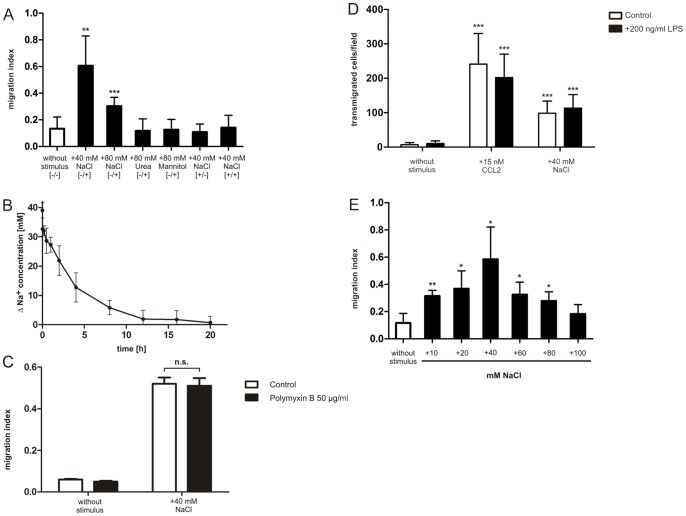
BMDMs and peritoneal macrophages, but no BMDCs show salt-dependent chemotaxis. Transwell migration assays of BMDMs (A), peritoneal macrophages (B) and BMDCs (C). The migration index was determined by the number of transmigrated cells in relation to cells transmigrated toward CXCL12 (A), CCL2 (B) or CCL19 (C), respectively. “+”indicates that the stimulus was added to the lower well of the transwell. Error bars indicate ± SD, n = 3, ***p<0.001 as compared to cells without stimulus.

In order to elucidate if primary macrophages also respond to excess NaCl as a chemoattractant, we investigated the migration behavior of murine bone marrow-derived macrophages (BMDMs) and macrophages isolated from the peritoneum of mice by the use of a transwell assay. We found in both BMDMs and peritoneal macrophages a significant migration response toward 40 mM excess NaCl. The migration capacity of BMDMs toward the NaCl stimulus was 75% as compared to the cells stimulated with the chemokine CCL2 (Fig. 2a), while peritoneal macrophages migrated about 50% as compared to CCL2-stimulated cells (Fig. 2b). Since the observed migration behavior was equivalent to the chemotactic response in RAW264.7 macrophages, we conclude that salt-dependent chemotaxis is not an isolated characteristic of a particular cell line.

We furthermore asked whether salt-dependent chemotaxis is a general feature of highly motile immune cells. Therefore, we analyzed the migration capacity of activated bone marrow-derived dendritic cells toward 40 mM excess NaCl. After stimulation with LPS dendritic cells mature into highly motile cells, which migrate toward the chemokine CCL19 in a transwell assay, but show no chemotactic response toward excess NaCl (Fig. 2c). Taken together, these results indicate that chemotaxis toward excess sodium is a macrophage specific process and not common in diverse motile myeloid cells.

### The actin cytoskeleton is not involved directly in early processes of NaCl-induced macrophage chemotaxis

**Figure 3:**
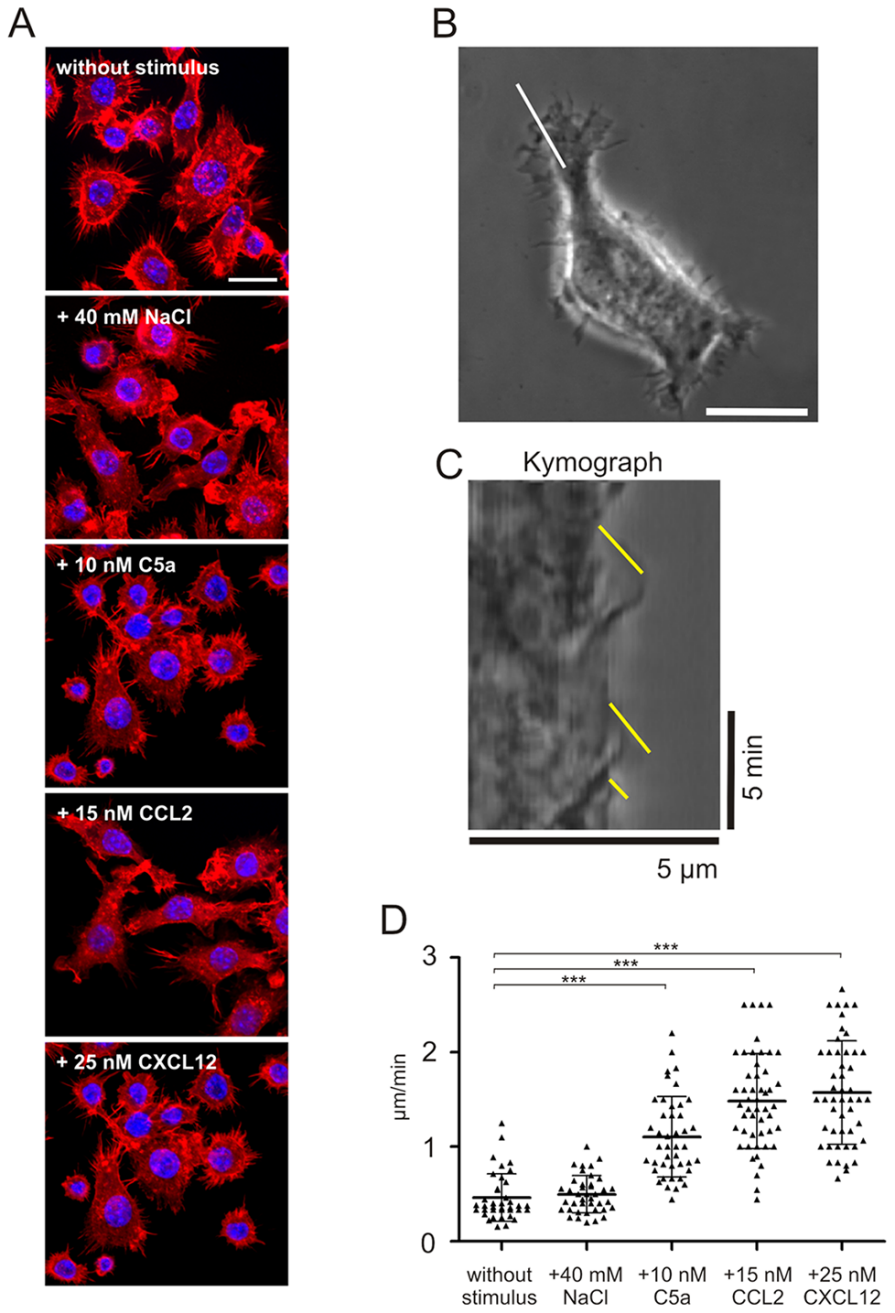
Excess NaCl does not increase lamellipodia dynamics of motile RAW264.7 cells. Microscopial analysis of TRITC-Phalloidin stained F-actin (red) in untreated RAW264.7 cells and cells stimulated with 40 mM NaCl, 10 nM C5a, 15 nM CCL2, or 25 nM CXCL12, respectively (A). Images were performed with an inverted confocal laser scanning microscope focussed to the basal plasma membrane of the cells. Nuclei were detected with DAPI staining (blue). Microscopical analysis of lamellipodia dynamics in RAW264.7 cells (B-D) on a glass surface stimulated with excess 40 mM NaCl, 10 nM C5a, 15 nM CCL2, 25 nM CXCL12, respectively. Membrane dynamics were visualized at the basal plasma membrane by the use of phase contrast over a period of 5 min at 2 sec per frame. Subsequently, an area of interest was marked on each image of the time-series by lines (white line in B) that cross the motile lamellipodium of the polarized cell. Velocities of lamellipodia protrusion formation were analyzed by kymograph analysis and line scan analysis of yellow lines (C) using ImageJ. Quantification of lamellipodia dynamics of motile RAW264.7 cells (D). Three kymographs per cell were analyzed; each dot represents the value of one single kymograph (C). Shown data are representative for one experiment out of three. Error bars indicate ± SD. ***p<0.001. Bars in microscopical images represent 10 µm (A, B).

Cell migration to chemoattractants highly depends on the dynamics of the actin cytoskeleton and the formation of polarized membrane protrusions (lamellipodia). We therefore analyzed the effects of excess NaCl on cell morphology, polarization and actin cytoskeleton in RAW264.7 cells. By the use of confocal laser scanning microscopy of phalloidin-stained macrophages we could not detect striking changes in cell morphology and lamellipodia formation following stimulation with NaCl, CXCL12, CCL2 or C5a, respectively (Fig. 3a). To analyze putative cytoskeletal differences in more detail, we used a more sensitive assay system that allowed us to follow the morphologic response of RAW264.7 cells by time-lapse phase contrast video microscopy. Velocity of membrane protrusion formation was analyzed by computer-assisted kymograph and line-scan analysis, using ImageJ software (Fig. 3b, c). Figure 3d shows that C5a, CCL2 and CXCL12 significantly increase velocities of membrane formation. While stimulation with CCL2 and CXCL12 resulted in a lamellipodia formation speed of about 1.5 µm/min, the presence of C5a resulted in a slower response with about 1 µm/min. Nevertheless, no significant differences in lamellipodia dynamics could be detected between unstimulated RAW264.7 cells and those treated with 40 mM excess NaCl (Fig. 3d). These data indicate that excess sodium is not involved directly in early events that increase actin dynamics and cytoskeletal reorganization of cell morphology.

### CCL2 and TonEBP are not involved in salt-dependent chemotaxis of macrophages

**Figure 4:**
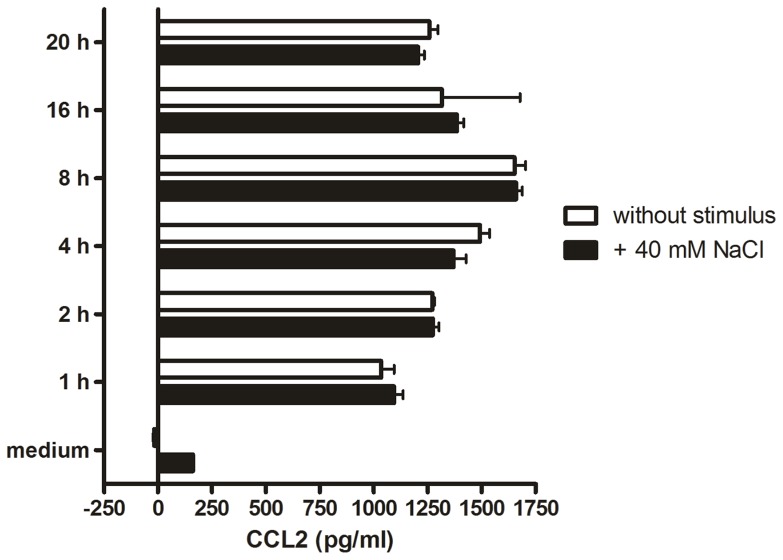
CCL2 production in RAW264.7 cells is not increased in excess NaCl. RAW264.7 cells were stimulated with additional 40

Since our results have shown that early migration-relevant processes like reorganization of the actin cytoskeleton were not altered under excess NaCl conditions in RAW264.7 cells, we investigated a possible induction of the chemokine CCL2 under hypertonic conditions. Studies by Kojima et al. had shown that NRK52E cells produced CCL2 in a time-dependent manner after exposure to hypertonicity by 100 mM NaCl and 200 mM mannitol [9]. For this reason, we measured CCL2 production in RAW 264.7 macrophages following stimulation with 40 mM excess NaCl over a time course of 20 hours. Enzyme linked immunosorbent assaying (ELISA) of the supernatants revealed significant CCL2 secretion of RAW264.7 macrophages, but no differences in CCL2 levels between unstimulated and NaCl-stimulated samples were observed at all time points investigated (Fig. 4). These results show that the secretion of CCL2 cannot be accounted for as a responsible indirect mechanism for salt-dependent chemotaxis in RAW264.7 cells.

**Figure 5:**
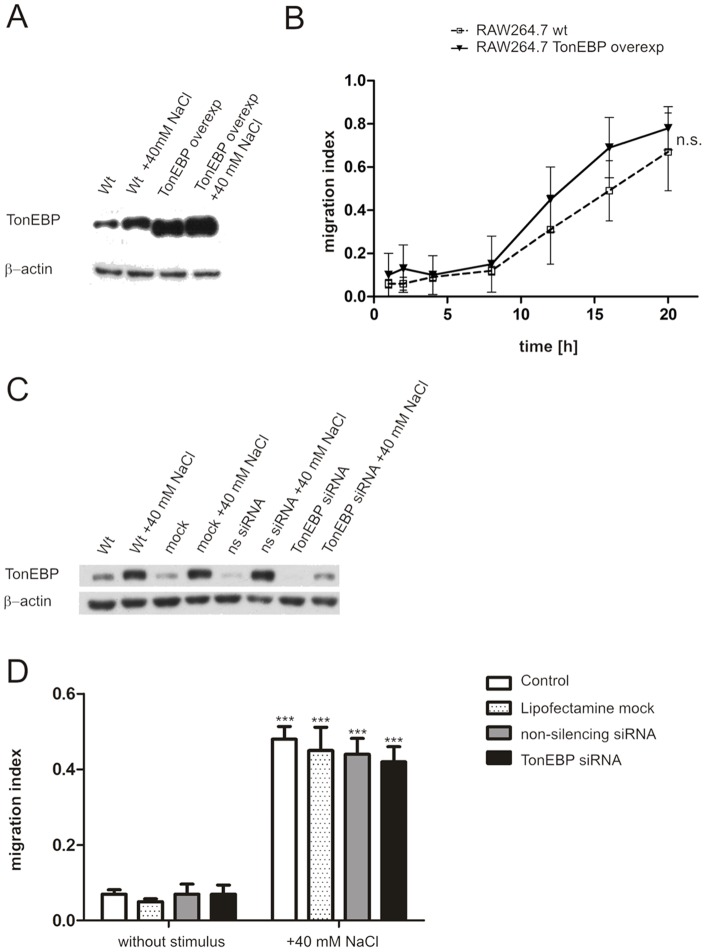
TonEBP is not involved in salt-dependent chemotaxis of macrophages. Western blot analysis of TonEBP protein expression with or without excess 40(wt) and TonEBP overexpressing (TonEBP overexp) RAW264.7 cells, respectively (A). Kinetics of cell migration of TonEBP overexpressing and wildtype RAW264.7 macrophages toward 40 mM excess NaCl over 20 hours using transwell migration assays (B). Western blot analysis of TonEBP expression in RAW264.7 cells following RNAi of TonEBP using lipofection for 72 hours; mock, lipofection without siRNA; ns siRNA, nonspecific control siRNA (C). Transwell migration assay of RAW264.7 cells following RNAi of TonEBP toward 40 mM excess NaCl (D). For all western blot analysis (A, C), β-actin protein expression was used as a loading control. The migration index (B, D) was determined by the number of transmigrated cells in relation to CXCL12-stimulated cells. “+”indicates that the stimulus was added to the lower well of the transwell (D). All cell migration data shown represent mean ± SD from 3 experiments performed in duplicate. ***p<0.001 as compared to cells without stimulus. n.s. not significant. n = 3.

Since other chemokines or chemotactically active factors might play important roles in this system, we assessed the role of the transcription factor tonicity-responsive enhancer binding protein (TonEBP), which protects cells from osmotic stress [10], [11], [12], [13] and which previously had been shown to control the transcription of chemokine genes, too [9], [14]. We thus investigated a possible role of TonEBP in salt-dependent chemotaxis using RAW264.7 macrophages with a stable TonEBP overexpression as described previously [2]. These cells display as high protein expression levels of TonEPB under normal culture conditions as wildtype cells show following stimulation with 40 mM excess NaCl (Fig. 5a). However, TonEBP protein levels did not increase further after stimulation with 40 mM excess NaCl in TonEBP overexpressing cells.

We further hypothesized that if TonEBP were responsible for salt-dependent chemotaxis, TonEBP overexpressing cells should show an increased migration response toward excess NaCl as compared to wild-type macrophages. However, a comparison of cell migration of wildtype RAW264.7 macrophages with TonEBP overexpressing cells over 20 hours revealed no significant differences in migration behavior (Fig. 5b). This finding was further corroborated by showing that the migratory capacity toward excess NaCl in RAW264.7 cells following RNAi of TonEBP (Fig. 5c and 5d) was normal. Taken together, these results indicate that TonEBP is not involved in NaCl-induced chemotaxis of macrophages.

## Discussion

Chemotaxis is an important process during an immune reaction in order to fight invading organisms. It is known that monocytes/macrophages are attracted to sites of infection by chemokines, bacterial components, leukotrienes, and complement factors [15], [16].

Interestingly, our results prompt us to propose the novel concept of salt-dependent chemotaxis of macrophages. We showed that macrophages of the cell line RAW264.7 interpret a high extracellular NaCl concentration as a chemoattractant and that they migrate actively toward this hypertonic stimulus. We furthermore found that this migration is directional and thus dependent on an increasing NaCl-gradient, since cells did not respond to the application of a reverse gradient or lack of a gradient by uniformly added excess NaCl. Since neither urea nor mannitol – increasing osmolality comparable to the response following NaCl – were able to induce cell migration, we concluded that hyperosmolarity or hypertonicity as a general origin of the migration response can be excluded. Apparently, salt-dependent chemotaxis was also characterized by a dose-dependency, with a maximum response at 40 mM excess NaCl. This concentration is in line with *in vivo* skin electrolyte differences of rats fed a high-salt diet [2]. At higher NaCl concentrations, it was impossible to separate the effects of a lower migration capacity from a simultaneous decrease in cell viability.

Salt-dependent chemotaxis is not restricted to RAW264.7 macrophage-like cells, since both murine BMDMs as well as peritoneal macrophages migrate toward excess NaCl. However, it is important to note here that not all motile cells of the myeloid lineage recognize a high NaCl concentration as a chemoattractant, since we could show that LPS-stimulated BMDCs do not migrate toward 40 mM excess NaCl. Although we cannot exclude that other motile cells show a chemotactic response toward a hypertonic NaCl stimulus, our results are consistent with the notion that salt-dependent chemotaxis is a macrophage-specific function.

How is extracellular NaCl sensed by RAW264.7 cells, and how does hypertonicity initiate directional cell migration? Our analysis of lamellipodia dynamics showed that NaCl was not capable of inducing early actin-dependent events. RAW264.7 cells stimulated with complement C5a, or with the chemokines CXCL12 or CCL2, respectively, displayed rapid actin-cytoskeletal reorganization and formation of lamellipodia in the first 30 minutes, but no significant difference in actin dynamics between unstimulated control and cells exposed to 40 mM excess NaCl was found. This indicates that excess NaCl has no direct effect on reorganization of the actin cytoskeleton.

It is therefore likely that other factors transduce the extracellular NaCl gradient signal into the cell to initiate indirect events, which eventually affect late chemotactic responses. One possible candidate late mechanism in salt-dependent chemotaxis might be the NaCl-induced expression of chemokines [9], [14], which are able to enhance cell motility in an autocrine/paracrine way, as described earlier for CCL2 in NRK52E cells [9]. However, we could not establish a role of excess NaCl in the induction of CCL2 in RAW264.7 cells. It needs to be stated that it is currently unclear whether NaCl induces the expression of other chemokines in this cell type. We speculate that chemokines with an autocrine/paracrine function could be secreted by a population of initially migrating cells followed by a delayed migration of remaining cells. This migration dynamic of “pioneering” cluster forming cells which later attract a massive influx of cells into that cluster has been described for neutrophils swarms during infection [17], [18]. These recent studies have shown that an autocrine/paracrine stimulation of the respective cells with chemoattractants, e.g. cytokines or leukotriene B4, is responsible for this “pioneering” migration and leads to an amplification of the initial chemoattractive signal.

It is further known that hypertonicity induced by NaCl leads to the expression of TonEBP, the only known mammalian transcription factor protecting cells against hypertonic stress [10], [11]. Upon activation, TonEBP enhances expression of several osmoprotective genes such as AR and SMIT to ensure an accumulation of organic osmolytes in the cell, or Hsp70 to stabilize proteins [12], [13]. Furthermore, a variety of other functions of TonEBP has been described, emphasizing its role in cell differentiation and migration. Jauliac et al. showed that TonEBP led to an increased chemotactic cell migration and invasion in human breast and colon carcinoma cell lines [19], while inhibition of TonEBP impaired migration and differentiation of cultured myoblasts [20]. Beyond that, it was recently shown that TonEBP-deficiency also reduced migration toward M-CSF in BMDMs [21].

Importantly, we could not establish a role of TonEBP in the regulation of NaCl-induced macrophage migration, because cell motility is unaltered in RAW264.7 cells following RNAi of TonEBP or in TonEBP-overexpressing cells, respectively.

In conclusion, we discovered that RAW264.7 macrophages are able to interpret excess NaCl as a chemoattractant, and future studies will have to address the precise underlying mechanism in detail. Altogether, our results may help to explain previous findings from Machnik et al. [2], in which macrophages accumulated in the skin of rats fed on a high salt diet. Since macrophages orchestrate interstitial electrolyte composition, it is important for these cells to accumulate, to sense and to react to a high interstitial salt concentration. Deficiencies in any of these functions might contribute to the development of hypertension, as previous results have suggested [2], [5].

## Supporting Information

Figure S1 **LPS-induced up-regulation of CD86 in BMDMs.** Analysis of CD86 expression in LPS-activated BMDMs using flow cytometry.
(TIF)

Figure S2 **RAW264.7 macrophages are viable in additional 40 mM NaCl.** Cell titer Blue viability assay measuring the metabolic activity after 20 hours exposure to excess 10 to 200 mM NaCl in serum-reduced media (DMEM 0.5% FCS). Cell viability index determined by comparing NaCl-stimulated to untreated control is shown as mean ± SD from 5 experiments performed in triplicate. *p<0.05, **p<0.01 as compared to control of untreated cells.
(TIF)

Table S1 **Osmolality of hypertonic stimuli determined by osmometer analysis.**
(TIF)

## References

[1] Murray PJ, Wynn TA (2001) Protective and pathogenic functions of macrophage subsets. Nat Rev Immunol 11: 723–37 doi:10.1038/nri3073 10.1038/nri3073 PMC3422549 21997792

[2] Machnik A, Neuhofer W, Jantsch J, Dahlmann A, Tammela T, et al (2009) Macrophages regulate salt-dependent volume and blood pressure by a vascular endothelial growth factor-C-dependent buffering mechanism. Nat Med 15: 545–52 doi:10.1038/nm.1960 19412173 10.1038/nm.1960

[3] Gerzer R, Heer M (2005) Regulation of body fluid and salt homeostasis-from observations in space to new concepts on Earth. Curr Pharm Biotechnol 6: 299–304. 16101468 10.2174/1389201054553662

[4] Titze J, Shakibaei M, Schafflhuber M, Schulze-Tanzil G, Porst M, et al (2004) Glycosaminoglycan polymerization may enable osmotically inactive Na+ storage in the skin. Am J Physiol Heart Circ Physiol 287: H203–H208. 14975935 10.1152/ajpheart.01237.2003

[5] Slagman MC, Kwakernaak AJ, Yazdani S, Laverman GD, van den Born J, et al (2012) Vascular endothelial growth factor C levels are modulated by dietary salt intake in proteinuric chronic kidney disease patients and in healthy subjects. Nephrol Dial Transplant 27: 978–82 doi:10.1093/ndt/gfr402 21778278 10.1093/ndt/gfr402

[6] Quast T, Eppler F, Semmling V, Schild C, Homsi Y, et al (2011) CD81 is essential for the formation of membrane protrusions and regulates Rac1-activation in adhesion-dependent immune cell migration. Blood 118: 1818–1827 doi:10.1182/blood-2010-12-326595 21677313 10.1182/blood-2010-12-326595

[7] Carpenter AE, Jones TR, Lamprecht MR, Clarke C, Kang IH, et al (2006) CellProfiler: image analysis software for identifying and quantifying cell phenotypes. Genome Biol 7: R100. 17076895 10.1186/gb-2006-7-10-r100 PMC1794559

[8] Tajima T, Murata T, Aritake K, Urade Y, Hirai H, et al (2008) Lipopolysaccharide induces macrophage migration via prostaglandin D2 and prostaglandin E2. J Pharmacol Exp Ther 326: 493–501 doi:10.1124/jpet.108.137992 18492946 10.1124/jpet.108.137992

[9] Kojima R, Taniguchi H, Tsuzuki A, Nakamura K, Sakakura Y, et al (2010) Hypertonicity-induced expression of monocyte chemoattractant protein-1 through a novel cis-acting element and MAPK signaling pathways. J Immunol 184: 5253–62 doi:10.4049/jimmunol.0901298 20368270 10.4049/jimmunol.0901298

[10] Miyakawa H, Woo SK, Dahl SC, Handler JS, Kwon HM (1999) Tonicity-responsive enhancer binding protein, a rel-like protein that stimulates transcription in response to hypertonicity. Proc Natl Acad Sci U S A 96: 2538–42. 10051678 10.1073/pnas.96.5.2538 PMC26820

[11] Ko BC, Turck CW, Lee KW, Yang Y, Chung SS (2000) Purification, identification, and characterization of an osmotic response element binding protein. Biochem Biophys Res Commun 270: 52–61. 10733904 10.1006/bbrc.2000.2376

[12] Takenaka M, Preston AS, Kwon HM, Handler JS (1994) The tonicity-sensitive element that mediates increased transcription of the betaine transporter gene in response to hypertonic stress. J Biol Chem 269: 29379–81. 7961914

[13] López-Rodríguez C, Aramburu J, Jin L, Rakeman AS, Michino M, et al (1999) NFAT5, a constitutively nuclear NFAT protein that does not cooperate with Fos and Jun. Proc Natl Acad Sci U S A 96: 7214–19. 10377394 10.1073/pnas.96.13.7214 PMC22056

[14] Kostyk AG, Dahl KM, Wynes MW, Whittaker LA, Weiss DJ, et al (2006) Regulation of chemokine expression by NaCl occurs independently of cystic fibrosis transmembrane conductance regulator in macrophages. Am J Pathol 169: 12–20. 16816357 10.2353/ajpath.2006.051042 PMC1698750

[15] Mantovani A, Sica A, Sozzani S, Allavena P, Vecchi A, et al (2004) The chemokine system in diverse forms of macrophage activation polarization. Trends Immunol 25: 677–86. 15530839 10.1016/j.it.2004.09.015

[16] Jones GE (2000) Cellular signaling in macrophage migration and chemotaxis. J Leukoc Biol 68: 593–602. 11073096

[17] Chtanova T, Schaeffer M, Han SJ, van Dooren GG, Nollmann M, et al (2008) Dynamics of neutrophil migration in lymph nodes during infection. Immunity 29: 487–496. 18718768 10.1016/j.immuni.2008.07.012 PMC2569002

[18] Lämmermann T, Afonso PV, Angermann BR, Wang JM, Kastenmüller W, et al.. (2013) Neutrophil swarms require LTB4 and integrins at sites of cell death in vivo. doi:10.1038/nature12175. 10.1038/nature12175 PMC3879961 23708969

[19] Jauliac S, López-Rodriguez C, Shaw LM, Brown LF, Rao A, et al (2002) The role of NFAT transcription factors in integrin-mediated carcinoma invasion. Nat Cell Biol 4: 540–44. 12080349 10.1038/ncb816

[20] O'Connor RS, Mills ST, Jones KA, Ho SN, Pavlath GK (2007) A combinatorial role for NFAT5 in both myoblast migration and differentiation during skeletal muscle myogenesis. J Cell Sci 120: 149–59. 17164296 10.1242/jcs.03307

[21] Halterman JA, Kwon HM, Leitinger N, Wamhoff BR (2012) NFAT5 expression in bone marrow-derived cells enhances atherosclerosis and drives macrophage migration. Front Physiol 3: 313 doi:10.3389/fphys.2012.00313 22934063 10.3389/fphys.2012.00313 PMC3429083

