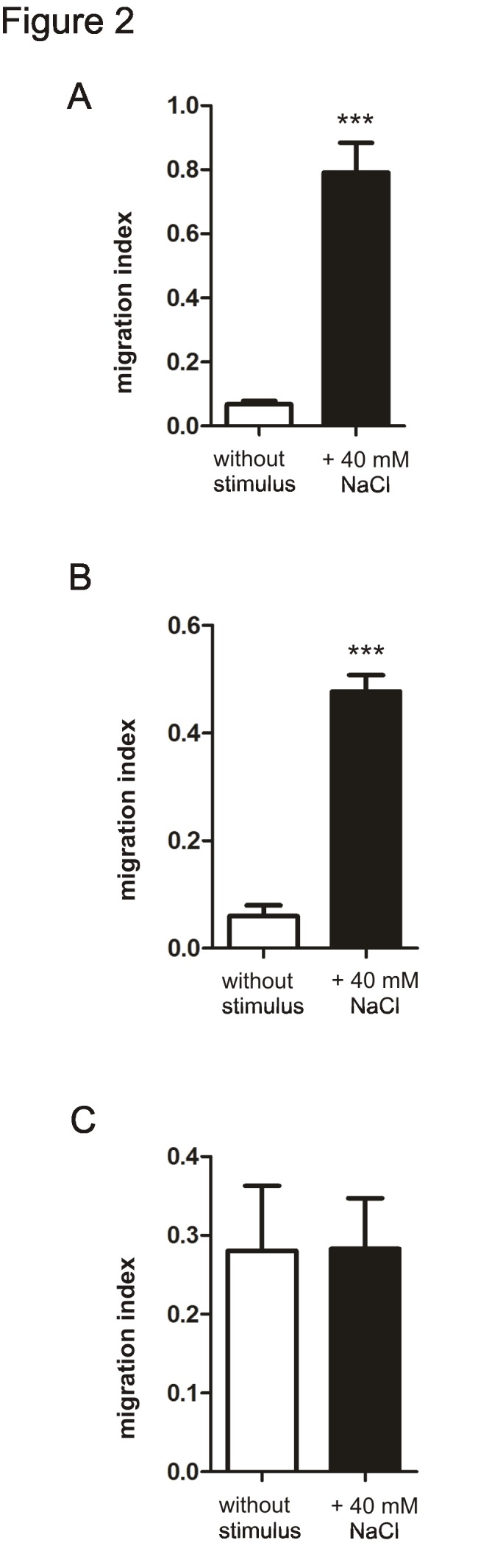# Correction: Salt-Dependent Chemotaxis of Macrophages

**DOI:** 10.1371/annotation/1907a0e9-290a-4221-a3fa-9147cb48a6f3

**Published:** 2013-10-30

**Authors:** Silke Müller, Thomas Quast, Agnes Schröder, Stephanie Hucke, Luisa Klotz, Jonathan Jantsch, Rupert Gerzer, Ruth Hemmersbach, Waldemar Kolanus

The version of Figure 2 that appeared in the paper was a duplicate of Figure 1. The correct version of Figure 2 is: 

**Figure:**