# Correction: Salt-Dependent Chemotaxis of Macrophages

**DOI:** 10.1371/journal.pone.0092756

**Published:** 2014-03-07

**Authors:** The PLOS ONE Staff

Figure 3 is incorrect. The authors have provided a corrected version here.

**Figure 3:**
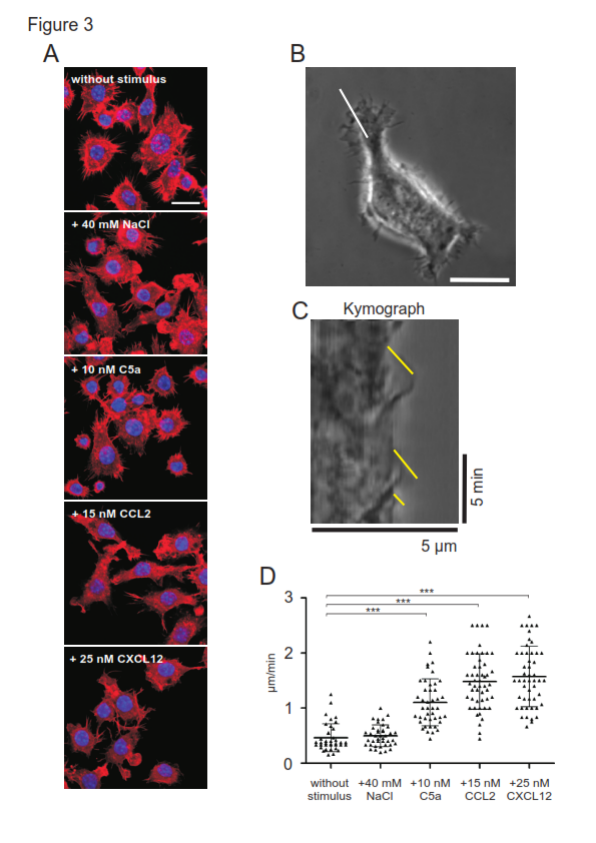
Excess NaCl does not increase lamellipodia dynamics of motile RAW264.7 cells. Microscopial analysis of TRITC-Phalloidin stained F-actin (red) in untreated RAW264.7 cells and cells stimulated with 40 mM NaCl, 10 nM C5a, 15 nM CCL2, or 25 nM CXCL12, respectively (A). Images were performed with an inverted confocal laser scanning microscope focussed to the basal plasma membrane of the cells. Nuclei were detected with DAPI staining (blue). Microscopical analysis of lamellipodia dynamics in RAW264.7 cells (B-D) on a glass surface stimulated with excess 40 mM NaCl, 10 nM C5a, 15 nM CCL2, 25 nM CXCL12, respectively. Membrane dynamics were visualized at the basal plasma membrane by the use of phase contrast over a period of 5 min at 2 sec per frame. Subsequently, an area of interest was marked on each image of the time-series by lines (white line in B) that cross the motile lamellipodium of the polarized cell. Velocities of lamellipodia protrusion formation were analyzed by kymograph analysis and line scan analysis of yellow lines (C) using ImageJ. Quantification of lamellipodia dynamics of motile RAW264.7 cells (D). Three kymographs per cell were analyzed; each dot represents the value of one single kymograph (C). Shown data are representative for one experiment out of three. Error bars indicate ± SD. ***p<0.001. Bars in microscopical images represent 10 μm (A, B).
