# Supplementary figures and images for: Salt-Dependent Chemotaxis of Macrophages
Source: PLoS One. 2013 Sep 16;8(9):e73439. doi: 10.1371/journal.pone.0073439 (PMC3774673; doi:10.1371/journal.pone.0073439)

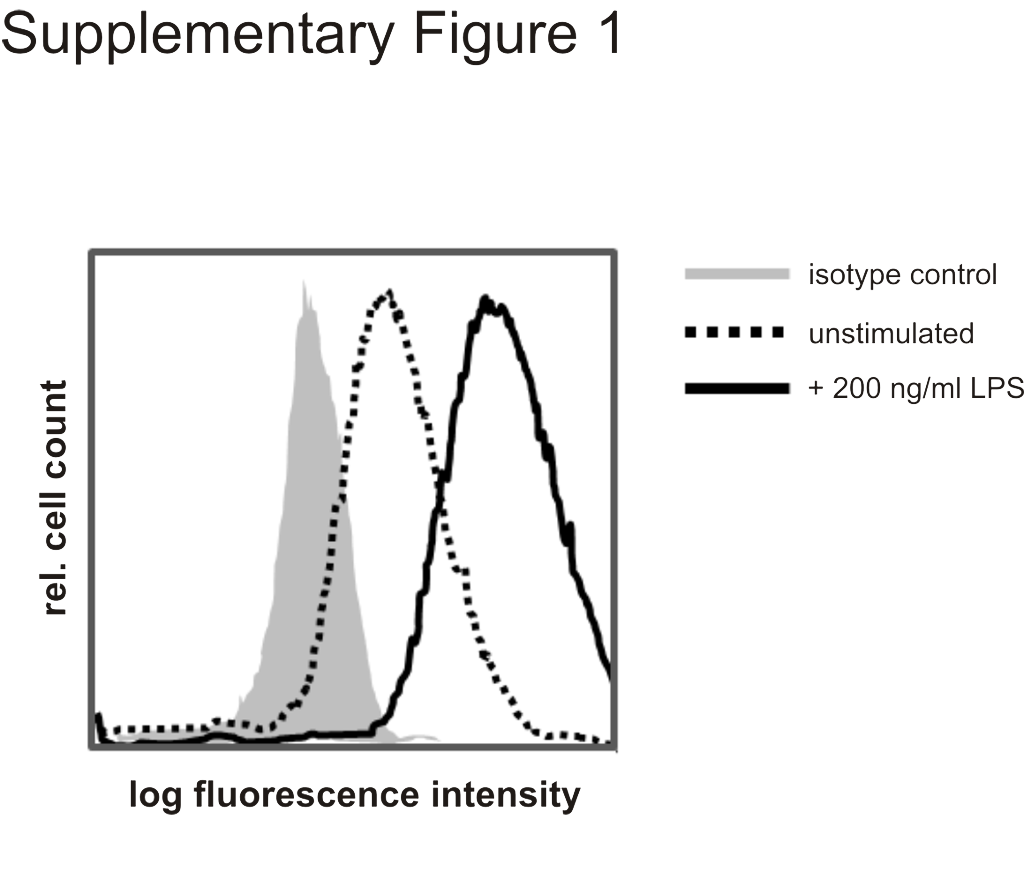

Supplement: Figure S1 — LPS-induced up-regulation of CD86 in BMDMs. Analysis of CD86 expression in LPS-activated BMDMs using flow cytometry. (TIF) [file pone.0073439.s001.tif]

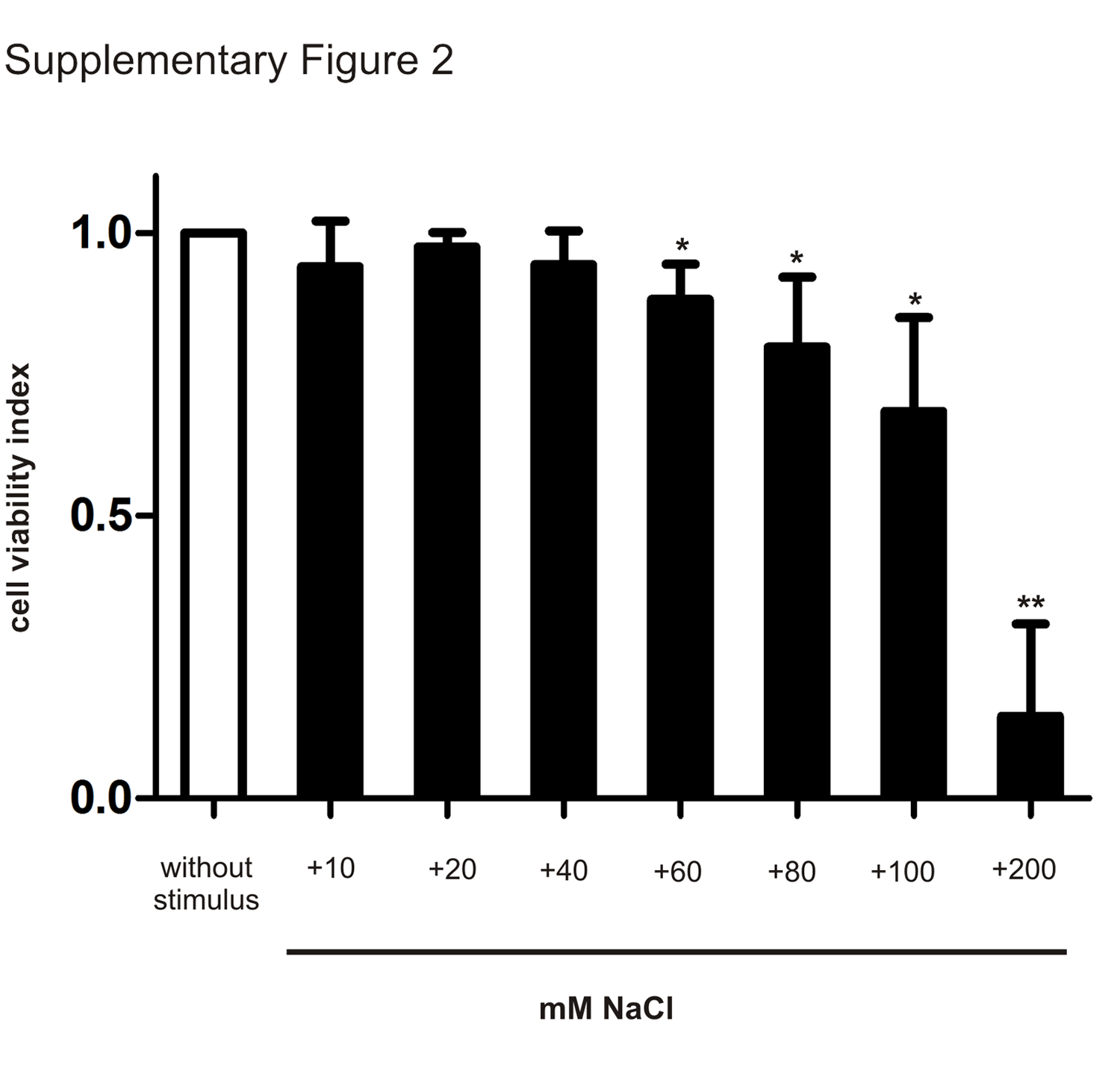

Supplement: Figure S2 — RAW264.7 macrophages are viable in additional 40 mM NaCl. Cell titer Blue viability assay measuring the metabolic activity after 20 hours exposure to excess 10 to 200 mM NaCl in serum-reduced media (DMEM 0.5% FCS). Cell viability index determined by comparing NaCl-stimulated to untreated control is shown as mean ± SD from 5 experiments performed in triplicate. *p<0.05, **p<0.01 as compared to control of untreated cells. (TIF) [file pone.0073439.s002.tif]

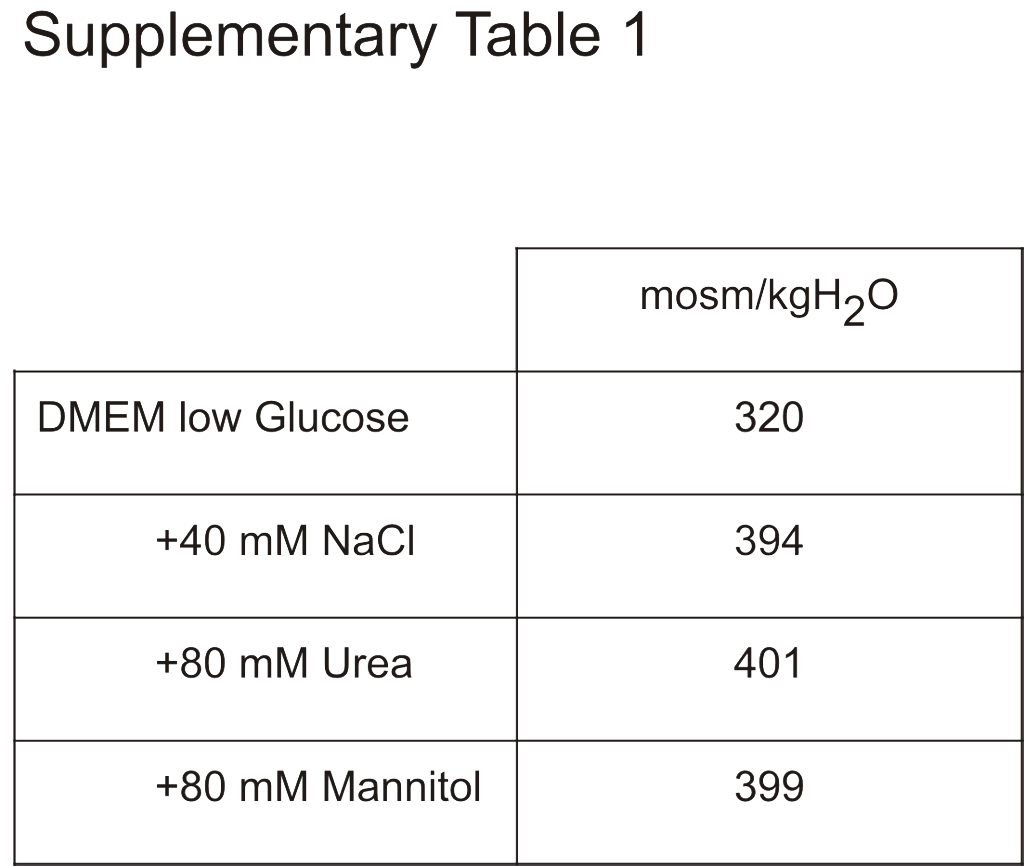

Supplement: Table S1 — Osmolality of hypertonic stimuli determined by osmometer analysis. (TIF) [file pone.0073439.s003.tif]
